# Tattoo-associated toxic shock syndrome: a case report

**DOI:** 10.1186/s12245-025-00993-2

**Published:** 2025-10-01

**Authors:** Takuya Kubo, Tetsuya Yumoto, Hideharu Hagiya, Koji Iio, Hiromichi Naito, Atsunori Nakao

**Affiliations:** 1 Department of Emergency, Critical Care, and Disaster Medicine, Faculty of Medicine, Dentistry, and Pharmaceutical Sciences, Okayama University, 2-5-1 Shikata-cho, Kita-ku Okayama, Okayama, 700-8558 Japan; 2 Department of Infectious Diseases, Okayama University Hospital, 2-5-1 Shikata-cho, Kita-ku Okayama, Okayama, 700-8558 Japan; 3 Microbiology Division, Clinical Laboratory, Okayama University Hospital, 2-5-1 Shikata-cho, Kita-ku Okayama, Okayama, 700-8558 Japan

**Keywords:** Blood culture, Critically ill, Septic shock, Tattooing, Toxic shock syndrome

## Abstract

**Background:**

Toxic shock syndrome (TSS) is a rare but life-threatening complication occasionally reported after tattooing.

**Case presentation:**

: A 29-year-old Japanese man was admitted to Okayama University Hospital, Okayama, Japan, in early spring 2025, one week after receiving a tattoo on his right shoulder and upper arm in Osaka. He presented with fever, gastrointestinal symptoms, hypotension, and multi-organ failure. Despite a failure to isolate a causative pathogen, he met clinical criteria for TSS. Supportive care and broad-spectrum antibiotics led to full recovery.

**Conclusions:**

TSS can occur after tattooing, even in individuals without apparent immunodeficiency. Pathogenic organisms may be unidentifiable; however, clinical diagnosis should not be delayed, and early therapeutic interventions are essential to improve outcomes.

## Background

Toxic shock syndrome (TSS) is a rare, life-threatening condition characterized by acute-onset high fever accompanying multi-organ involvement, including rash, vomiting, diarrhea, impaired consciousness, and circulatory failure. The fatal disease is commonly evoked by toxic shock syndrome toxin-1 (TSST-1) producing *Staphylococcus aureus* [1]. Importantly, the diagnosis of TSS should be based on clinically standardized diagnostic criteria, rather than laboratory confirmation of the specific exotoxin, which facilitates expeditious diagnosis and appropriate therapeutic intervention without unnecessary delays [2].

Historically, menstrual TSS was the representative condition of the disease, which has declined significantly in its incidence largely due to the decreased use of high-absorbency tampons. Today, the majority of the identifiable TSS cases are of non-menstrual origin, occurring in diverse clinical settings including post-surgical infections, postpartum complications, burn injuries, and skin or soft tissue infections [3]. These cases involve a broader demographic spectrum, including both pediatric and geriatric populations without sex predilection. Tattoo-associated TSS cases have been documented in the literature, typically manifesting within a two-week period following tattoo application under poor hygienic conditions with contaminated ink [4]. Such tragic cases are known to develop in healthy individuals without underlying risk factors [4]. However, there is limited literature addressing the diagnostic challenges and clinical implications of isolating coagulase-negative staphylococci (CoNS) in tattoo-associated TSS, particularly when contamination is not evident, and in patients with comorbidities such as alcoholic hepatitis.

Herein, we present the case of a young Japanese man who developed a clinical presentation consistent with TSS one week after receiving a tattoo. This case highlights that cosmetic tattooing can potentially precipitate TSS, even in individuals without an obvious immunocompromising condition, although pre-existing conditions such as alcoholic hepatitis may increase the vulnerability to the fatal disease.

### Case presentation

#### Patient information

A 29-year-old Japanese man with a history of alcoholic hepatitis was transported to Okayama University Hospital, Okayama, Japan, in early spring 2025 with a three-day history of fever, chills, vomiting, and diarrhea. He had received a tattoo in Osaka on his right shoulder and upper arm one week prior—the twelfth time he had been tattooed by the same artist. The day before admission, he visited a local outpatient clinic and was diagnosed with acute enterocolitis.

#### Clinical findings

**Fig. 1:**
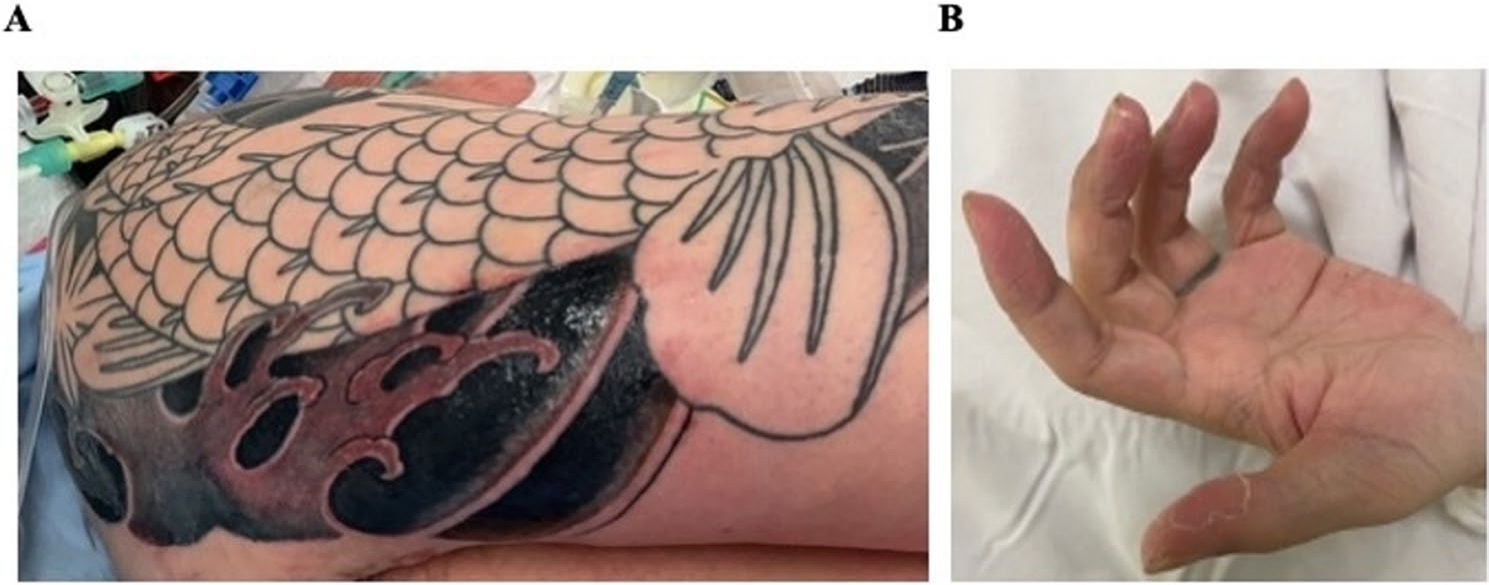
Non-purulent discharge is visible at the site of the recent tattoo on the patient’s right upper arm **A** and characteristic desquamation of the left fingertips observed on day 5 **B**

On arrival, he appeared pale, distressed, and exhibited pronounced whole-body rigors. His vital signs were as follows: Glasgow Coma Scale score, 15 (E4V5M6); blood pressure, 82/41 mmHg; pulse rate, 123 beats/min; respiratory rate, 30 breaths/min; and body temperature, 39.0 °C. Physical examination revealed bilateral conjunctivitis, mild erythematous rash on the cervical region, and non-purulent discharge from the site of the recent tattoo (Fig. 1A). No obvious evidence of necrotizing fasciitis was observed on the right upper extremity.

#### Diagnostic assessment

Arterial blood gas analysis revealed severe metabolic acidosis (pH 7.263 with base excess at −14.0 mmol/L) with an elevated lactate level (5.9 mmol/L). Laboratory tests indicated a progressive inflammatory state (White blood cell count, 15,670/µL; C-reactive protein, 15.7 mg/dL; and Procalcitonin, 146.4 ng/mL) as well as acute multi-organ failures (Table 1). A non-contrast enhanced computed tomography scan from the chest to pelvis revealed mild distention and edema of the small bowel and colon, suggestive of enterocolitis.

**Table 1:** Laboratory data on arrival

Blood gas analysis (F_I_O_2_ of 0.5)	Value	Refernece range	Unit		Biochemistry	Value	Refernece range	Unit
pH	7.263	7.35–7.45			Total protein	5.2	6.6–8.1	g/dL
PaCO_2_ (mmHg)	26.2	35.0–45.0	mmHg		Albumin	2.8	4.1–5.1	g/dL
PaO_2_	172	> 80	mmHg		AST	109	13–30	U/L
Base excess	−14.0	−3.3 to 2.3	mmol/L		ALT	118	10–42	U/L
Lactate	5.9	< 1.0	mmol/L		Total bilirubin	3.04	0.40–1.50	mg/dL
Complete blood count					LDH	436	124–222	U/L
White blood cell	15,670	3,300-8,600	/µL		BUN	44.5	8.0–20.0	mg/dL
Neutrophils	95.7	38.5–80.5	%		Creatinine	5.81	0.65–1.07	mg/dL
Hemoglobin	16.5	13.7–16.8	g/dL		Sodium	131	138–145	mmol/L
Hematocrit	47.4	40.7–50.1	%		Potassium	4.8	3.6–4.8	mmol/L
Platelet	177	158–348	*10^3/µL		Chloride	102	101–108	mmol/L
Coagulation					Glucose	83	73–109	mg/dL
PT	35	73–118	%		C-reactive protein	15.7	< 0.15	mg/dL
PT-INR	1.72	0.80–1.20			Procalcitonin	146.4	< 0.05	ng/mL
APTT	116.5	24–34	s					
Fibrinogen	370	200–400	mg/dL					
D-dimer	18.5	< 0.5	µg/mL					

F_I_O_2_ fraction of inspired oxygen, PaCO_2_ partial pressure of carbon dioxide, PaO2 partial pressure of oxygen, P prothrombin time, INR international normalized ratio, APTT activated partial thromboplastin time, AST aspartate aminotransferase, ALT alanine transaminase, LDH lactate dehydrogenase,BUN blood urea nitrogen

#### Therapeutic intervention

**Fig. 2:**
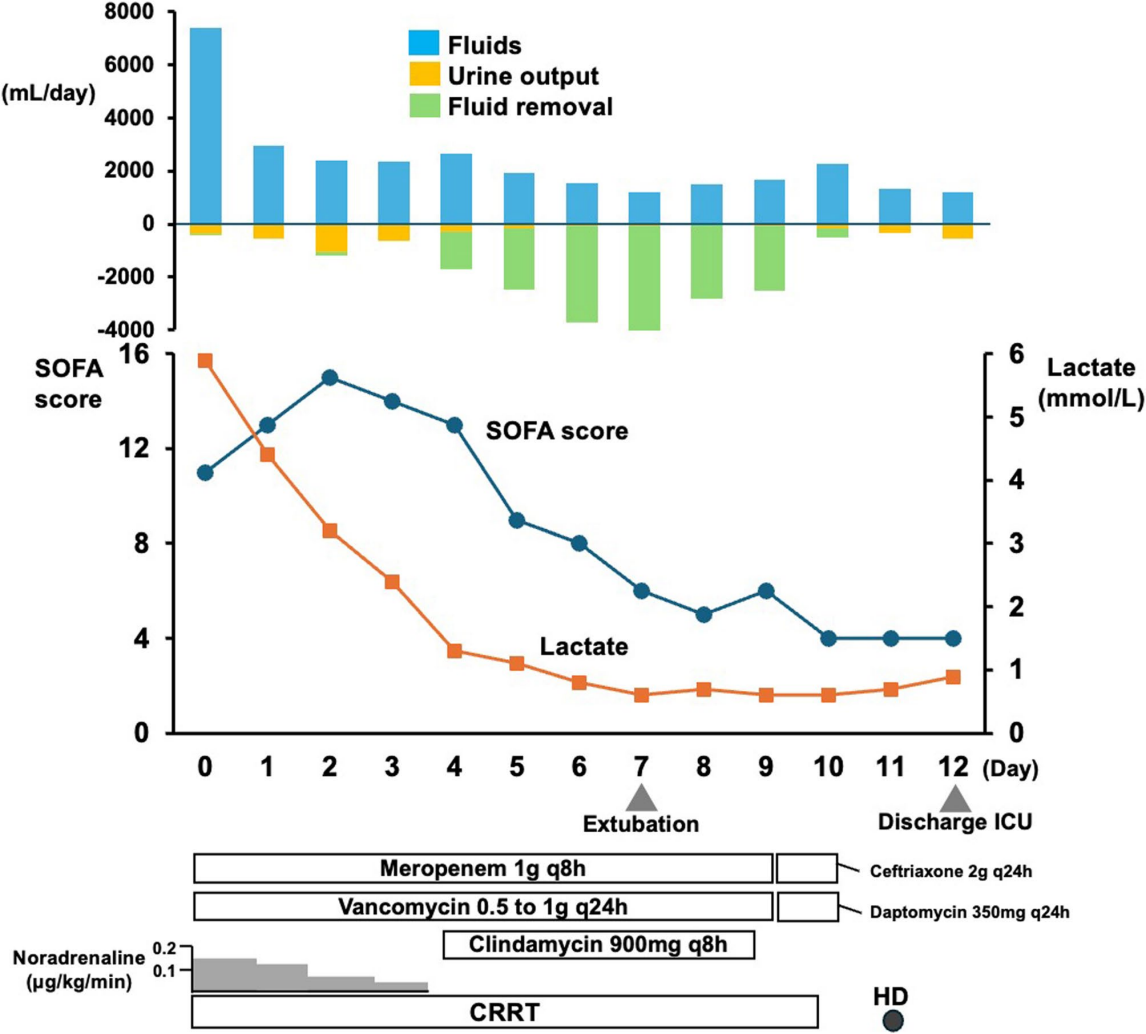
Clinical course of the patient including daily fluid balance CRRT: continuous renal replacement therapy, HD: hemodialysis, SOFA: Sequential Organ Failure Assessment, ICU: intensive care unit

Under the diagnosis of septic shock, fluid resuscitation was immediately initiated along with an empirical antibiotic treatment with meropenem and vancomycin. Upon admission to the intensive care unit (ICU), the patient remained hypotensive despite resuscitation and was therefore intubated and mechanically ventilated. Continuous renal replacement therapy was also introduced for anuria and persistent metabolic acidosis, with a blood flow rate of 100 mL/min and a filtration volume of 25mL/kg/h. Six units of fresh frozen plasma were administered for coagulopathy, resulting in an improvement of INR from 1.72 to 1.19 by the following day, after which no further fresh frozen plasma was required. Liver function tests showed a decrease in aspartate aminotransferase and alanine transaminase levels to 89 U/mL and 93 U/mL, respectively, by the following day. Serum bilirubin peaked at 4.48 mg/dL on the following day and subsequently improved. Blood cultures drawn on the day of admission detected *Staphylococcus capitis* in one of two sets, with growth only in the aerobic bottle after an incubation period of 19.7 h. While this finding could represent contamination, a potential role in the pathogenesis of TSS could not be excluded. Therefore, we tested the isolate at our hospital laboratory for the presence of the TSST-1 gene and Panton-Valentine leukocidin activity and found both to be absent. A skin swab specimen was collected from the tattoo site, but no bacterial growth was observed. On day 5, characteristic desquamation was observed at digital extremities (Fig. 1B). Based on the clinical presentation, progression, and exclusion of other infectious etiologies, a definitive diagnosis of TSS was established. Intravenous clindamycin was additively administered to suppress toxin production in suspected TSS, and the patient was weaned off mechanical ventilation and extubated on day 7. Antimicrobial therapy was changed to a combination regimen of ceftriaxone and daptomycin on day 9 to maintain broad coverage while reducing potential nephrotoxicity associated with vancomycin, in consideration of the patient’s persistent renal dysfunction. Following a 10-day course of antimicrobial therapy, the patient was discharged from the ICU on day 12 (Fig. 2).

#### Follow-up and outcomes

He received his final session of hemodialysis on day 13, after which his renal function improved, and further dialysis was no longer required. On day 17, he was transferred to another hospital for rehabilitation and finally discharged on day 23 with a normalized serum creatinine level of 1.03 mg/dL.

#### Patient perspective

After discharge, the patient reported a complete return to his normal daily life and stated that he would never undergo tattooing again.

## Discussion

This case underscores two key clinical insights. First, TSS can occur as a complication of tattooing even when no overt procedural lapses are identified. Second, a clinical diagnosis of TSS is achievable based on characteristic clinical manifestations according to established diagnostic criteria, even in the absence of identified etiological pathogens.

TSS and necrotizing fasciitis are among the most severe but exceedingly rare complications of tattooing​ [5–7]. Most reported cases of tattoo-associated TSS have reportedly involved clear lapses in aseptic technique or traditional practices with potential contamination [4]. In our case, the patient had been tattooed multiple times previously by the same artist without complications. However, in Japan, no licensing system exists for tattoo parlors, and a breakdown in hygienic processes during the patient’s most recent tattoo session may have predisposed him to the fatal infection. The absence of detailed procedural documentation, inspection of the facility, or verification of ink sterility precluded confirmation of fully sterile practice. In addition, the patient’s history of alcoholic hepatitis may have increased his vulnerability to infection. This case therefore highlights the risk of severe infection, including TSS, following tattooing in individuals with underlying comorbidities, even when the procedure appears uneventful and no procedural lapses are identified. Clinicians should maintain a high index of suspicion for TSS in patients presenting with acute-onset shock and multi-organ dysfunctions shortly following tattooing, regardless of the circumstances or professional background of the tattoo provider.

Tattooing can introduce the involvement of various pathogens, such as *S. aureus Streptococcus pyogenes*, and even *Pseudomonas* spp [8]. Rapidly growing mycobacteria, representatively *M. chelonae* and *M. abscessus*, also have the potential to cause outbreaks linked to contaminated tattoo inks, including those from unopened bottles [9]. Nontuberculous mycobacteria infections particularly require prolonged multidrug therapy, underscoring the importance of strict ink quality control and aseptic technique.

This case also highlights the clinical significance of making a diagnosis of TSS even when any causative pathogens remain unidentified through microbiological testing. According to the Centers for Disease Control and Prevention criteria, TSS is clinically diagnosed based on fever of ≥ 38.9 °C, diffuse rash with subsequent desquamation, hypotension, and involvement of at least three organ systems, after excluding other causes [10]​; an isolation of a pathogen is not required for the definitive diagnosis as in our case.

Pathogenic role of *S. capitis* isolated from blood culture should be discussed. The organism was detected in only one of two blood culture sets and thus was considered a contamination. CoNS are typically of low virulence and have rarely been implicated in severe infections; however, a case of CoNS-induced TSS has been documented [11]. While most strains lack TSST-1, some harbor superantigen-encoding genes and may trigger TSS-like cytokine storms via non-superantigen pathways [12, 13]. In our case, TSST-1 and Panton-Valentine leukocidin activities were absent, and the single positive blood culture limits causal inference; nevertheless, the temporal association with disease onset suggests a possible contributory role.

In suspected TSS, an empiric therapy should provide antimicrobial coverage for *S. aureus* (including MRSA) and *S. pyogenes*, with the addition of clindamycin to inhibit toxin production [14]. In cases with septic shock, the national guideline recommends empiric therapy with a broad-spectrum β-lactam (e.g., piperacillin-tazobactam or meropenem) plus vancomycin, as was done in this case, followed by de-escalation once culture and susceptibility results are available [15]. The role of adjunctive intravenous immunoglobulin (IVIG) in TSS remains debatable. In streptococcal TSS, a meta-analysis of clindamycin-treated cases showed reduced mortality [16], but a large multicenter study of necrotizing fasciitis with shock found no survival benefit, even with group A *Streptococcus* or *S. aureus* [17]. In our case, IVIG could have been considered, but its benefit is uncertain.

This case report has several limitations. The causative pathogen was not identified; an isolation of *S. capitis* from blood culture was considered a contamination rather than an active infection. We could not verify the hygienic standards of the tattoo procedure or determine the sterility of the tattoo ink used for the patient. Finally, as this is a single-patient case, the findings may not be generalizable, and causal inference remains limited.

In conclusion, this case demonstrates that tattooing can be complicated by rapidly progressive TSS in a patient with a history of alcoholic hepatitis. Early recognition with prompt antimicrobial and supportive therapy can lead to favorable outcomes.

## Data Availability

No datasets were generated or analysed during the current study.

## Abbreviations

CoNS: Coagulase-negative staphylococci
ICU: Intensive care unit
IVIG: Intravenous immunoglobulin
TSS: Toxic shock syndrome
TSST-1: Toxic shock syndrome toxin-1
